# 同位素稀释气相色谱-三重四极杆质谱法测定环境空气中的多氯萘

**DOI:** 10.3724/SP.J.1123.2021.12006

**Published:** 2022-07-08

**Authors:** Hongyuan LIU, Jing JIN, Cuicui GUO, Jiping CHEN, Chun HU

**Affiliations:** 1.沈阳药科大学, 辽宁 沈阳 110000; 1. Shenyang Pharmaceutical University, Shenyang 110000, China; 2.中国科学院分离分析化学重点实验室, 中国科学院大连化学物理研究所, 辽宁 大连 116023; 2. CAS Key Laboratory of Separation Science for Analytical Chemistry, Dalian Institute of Chemical Physics, Chinese Academy of Sciences, Dalian 116023, China; 3.中国科学院大学, 北京 100012; 3. University of Chinese Academy of Sciences, Beijing 100012, China

**Keywords:** 气相色谱-三重四极杆质谱, 同位素稀释, 多氯萘, 环境空气, gas chromatography-triple quadrupole mass spectrometry (GC-MS/MS), isotope dilution, polychlorinated naphthalenes (PCNs), ambient air

## Abstract

环境空气中的多氯萘(PCNs)一般为痕量水平(pg/m^3^),要实现其准确定量必然对分析方法的提取、净化和仪器分析提出较高要求。研究通过考察提取溶剂种类、净化流程和色谱-质谱参数,建立了加速溶剂萃取(ASE)-多层硅胶复合中性氧化铝柱的净化方法,并利用同位素稀释气相色谱-三重四极杆质谱(GC-MS/MS)对环境空气中的多氯萘进行测定。同时,通过在采样、提取和进样分析前分别添加同位素内标,开展质量控制和保证。结果表明,在2~100 ng/mL范围内3~8氯萘的平均相对响应因子(RRF)的相对标准偏差(RSD)均小于16%。PCNs同类物的方法检出限为1~3 pg/m^3^(以样品体积为288 m^3^计算)。采用基质加标法评价了方法对环境空气样品中PCNs测定的精密度和准确度,低、中、高加标水平下3 ~8氯萘的平均加标回收率分别为89.0%~119.4%、98.6%~122.5%和93.7%~124.5%,测定结果的平均相对标准偏差分别为1.9%~7.0%、1.6%~6.6%和1.0%~4.8%。整个分析过程中,采样内标和提取内标的平均回收率分别为136.2%~146.0%和42.4%~78.1%, RSD分别为5.6%~7.5%和2.7%~17.5%,满足痕量分析的要求且平行性较好。方法的灵敏度和准确度高,精密度良好,适用于环境空气中3~8氯萘的准确定量测定,可在一定程度上缓解多氯萘监测对高分辨气相色谱-高分辨质谱的依赖,为实现多氯萘的国际履约提供方法支持。

## Abstract (en)

Polychlorinated naphthalenes (PCNs) have a structure similar to that of polychlorinated biphenyls (PCBs) and represent a new type of persistent organic pollutants (POPs) that are widely present in the environment and biological communities. PCNs can migrate and transform via different environmental media, which severely affects the health of humans and organisms. Researchers have devoted considerable focus on ambient air pollution. Although the current ambient air quality has not yet limited the concentration of PCNs, the Stockholm Convention has required parties to prohibit and eliminate their production and use. As one of the contracting parties, China is obligated to improve its environmental monitoring. In other words, the development of a method for monitoring PCNs in ambient air is important for understanding ambient air quality and safeguarding human health. PCNs are generally present at trace levels (pg/m^3^) in ambient air. To achieve accurate quantification of PCNs, high demands are raised on the methods for extraction, purification, and instrumental analysis, which can directly affect the efficiency, accuracy, and sensitivity of a method.

Considering the trace-level presence of PCNs in ambient air and the high efficiency and accuracy of the analytical method, accelerated solvent extraction (ASE), combined with column chromatography using a multilayer silica gel column and a neutral alumina column, was established for the extraction and purification of PCNs in ambient air. The important parameters involved in the aforementioned steps, such as the type of extraction and volume of elution solvent, were optimized. The results indicated that dichloromethane-hexane (1:1, v/v) was the best extraction solvent for the recovery of PCNs. Hexane and dichloromethane-hexane (5:95, v/v) were used as the elution solvents for the multi-silica gel column and neutral alumina column, respectively. Isotope dilution gas chromatography-triple quadrupole mass spectrometry (GC-MS/MS) was used to quantify the target compounds. Gas chromatographic parameters, such as temperature program conditions and inlet temperature, were also optimized. The oven temperature program was as follows: 80 ℃ for 1 min, 80 ℃ to 160 ℃ at 15 ℃/min, 160 ℃ to 265 ℃ at 3 ℃/min, and 265 ℃ to 280 ℃ at 5 ℃/min, followed by holding the temperature at 280 ℃ for 10 min. The inlet temperature was set at 260 ℃. The optimal characteristics of ion pair, collision energy, and ion source temperature were determined by optimizing the key mass spectrometry parameters. The developed instrumental method, combined with suitable sample preparation techniques, was used to determine the concentrations of PCNs in ambient air samples. Quality control (QC) and quality assurance (QA) were performed by adding isotope internal standards before sampling, extraction, and injection analysis to monitor the entire analysis process. The relative standard deviations (RSDs) of the relative response factors (RRFs) for trichloronaphthalene to octachloronaphthalene were less than 16% in the concentration range of 2-100 ng/mL. The method detection limits (MDLs) for PCN homologues were in the range of 1-3 pg/m^3^(calculated using a sample volume of 288 m^3^). The precision and accuracy of this method for determining PCNs in ambient air samples were evaluated using a spiked matrix. The average spiked recoveries of trichloronaphthalene to octachloronaphthalene were 89.0%-119.4%, 98.6%-122.5% and 93.7%-124.5% at low, medium, and high spiked concentrations (20, 50, and 90 ng/mL), respectively. The RSDs of the assay results were 1.9%-7.0%, 1.6%-6.6%, and 1.0%-4.8%, respectively. During the entire analysis process, the average recoveries of the sampling and extracted internal standards were 136.2%-146.0% and 42.4%-78.1%, respectively, and the corresponding RSDs were 5.6%-7.5% and 2.7%-17.5%. Thus, this method meets the requirements of trace analysis and exhibits good parallelism, high sensitivity, high accuracy, and good precision, and it is suitable for the accurate quantitative determination of trichloronaphthalene to octachloronaphthalene in ambient air.

多氯萘(PCNs)是一类基于萘环上氢原子被氯原子取代的氯代芳环化合物(结构式见图1),化学通式为C_10_H_8-_*_n_*Cl*_n_*,共有75种同类物。PCNs具有良好的化学惰性、抗热性、绝缘性、防水性和阻燃性,被广泛用作阻燃剂、电容器和电压器的绝缘油、电缆绝缘体、燃料保护剂和木材杀菌剂等^[1]^。20世纪70年代起,全球范围内开始陆续停止PCNs的生产和使用。环境中PCNs的主要污染来源有早期的工业生产及应用、废弃物焚烧或金属冶炼等工业热过程的产生和排放,以及多氯联苯(PCBs)生产过程中产生的PCNs杂质^[2,3]^。PCNs具有类似二噁英类物质的毒性、生物累积性和持久性,不仅能够通过大气进行远距离传输^[4]^,而且可能在人类和动物体内引起严重甚至致命的生物效应^[5]^。正因为如此,PCNs已于2015年被正式列入《斯德哥尔摩公约》的受控名单。

**图 1:**
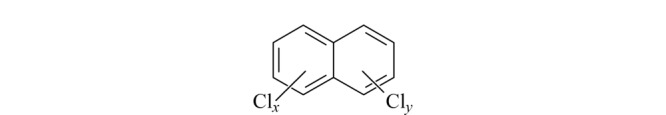
多氯萘的结构式

呼吸摄入作为人体PCNs暴露的重要途径之一,空气污染所带来的环境和人体健康问题受到普遍关注。目前,国内外尚未发布环境空气相关行业标准用来规范PCNs检测流程,因此开发适用于环境空气中多氯萘测定的准确、灵敏的分析方法,对于完善环境空气中多氯萘监测方法体系、满足国际履约具有重要意义。据报道,环境空气中PCNs含量一般在pg/m^3^水平^[6]^,且主要分布在气相中^[7⇓-9]^。毒性当量因子(TEF)通常被用于评价具有芳香烃受体活性的化学品的风险。Puzyn等^[10]^对比了不同测定系统,得出了PCNs的毒性当量因子值,其中1~2氯萘的TEF值比其他PCNs低1~7个数量级,对环境和人体潜在危害较低。因此,3~8氯萘通常被作为多氯萘的主要研究对象。

大气中PCNs的提取方法主要包括索式提取和加速溶剂萃取。索式提取是环境污染物分析中最常用的萃取方法,虽然该方法操作简单,萃取效率较高,但溶剂量消耗较大,萃取时间较长。相比而言,加速溶剂萃取则具有有机溶剂消耗量少、萃取时间短和可实现高通量样品萃取等特点,在环境分析领域广受关注^[11,12]^。由于复杂样品中共提取的脂类、硫化物和色素等物质会干扰PCNs的检测和定量,层析柱是目前使用最广泛的净化方法^[6,8,13]^。气相色谱-高分辨质谱(GC-HRMS)具有低检出限、高灵敏度和选择性,可实现目标物的准确定性定量,是PCNs最常用的仪器测定方法^[12,14,15]^,但是该仪器购置和维护成本较为昂贵,且对实验室条件和操作人员要求较高,仪器设备普及率不足。相比而言,气相色谱-串联质谱(GC-MS/MS)的运行成本相对较低,且具有较好的灵敏度和选择性^[6,16,17]^,近年来在环境中持久性有机污染物的检测方面发展迅速。

本研究根据《环境空气半挥发性有机物采样技术导则》(HJ 691-2014)和《环境监测分析方法标准修订技术指导》(HJ 168-2020)和国际上关于水质中多氯萘测定的技术规范(ISO TS 16780-2015),从样品采集、前处理优化和仪器检测进行整个流程的分析方法开发,采用加速溶剂萃取-多层硅胶柱复合中性氧化铝柱进行提取净化,同时结合同位素稀释气相色谱-三重四极杆质谱法,建立了环境空气样品中3~8氯萘的分析方法,并通过方法检出限、精密度和准确度试验对方法进行适用性验证。

## 1 实验部分

### 1.1 仪器、试剂与材料

Echo Hivol大流量主动采样仪(意大利Tcr Tecora公司); R-205旋转蒸发仪(瑞士Buchi公司); ASE-350加速溶剂萃取仪和TSQ Quantum XLS气相色谱-三重四极杆质谱仪(美国Thermo Fisher公司); DC-12氮吹浓缩仪(上海安普公司)。

丙酮、正己烷、二氯甲烷、壬烷(农残级,美国J. T. Baker公司);浓硫酸、氢氧化钾和无水硫酸钠(优级纯和分析纯,天津市大茂化学试剂厂);硝酸银(分析纯,沈阳试剂二厂);硅胶(100~200目,青岛恒泽硅胶制药有限公司);中性氧化铝(pH=7.5,美国MP Biomedicals公司);聚氨基甲酸酯泡沫(高:50 mm,直径:90 mm,密度:28 mg/m^3^,北京赛富莱博科技有限公司); MK360石英纤维滤膜(203×254 mm,瑞典Munktell Filter公司)多氯萘工业产品标准溶液(Halowax 1000和1014)购自美国AccuStandard公司;10种PCNs标准品(包括CN-24、CN-13、CN-42、CN-46、CN-52、CN-53、CN-66、CN-68、CN-73、CN-75)和10种^13^C标记的PCNs标准品(包括^13^C_10_-CN-2、^13^C_10_-CN-13、^13^C_10_-CN-42、^13^C_10_-CN-27、^13^C_10_-CN-52、^13^C_10_-CN-67、^13^C_10_-CN-64、^13^C_10_-CN-65、^13^C_10_-CN-73、^13^C_10_-CN-75)均购自美国Cambridge Isotope Laboratories;6种 ^13^C 标记的PCBs标准品(包括^13^C_12_-PCB-28、^13^C_12_-PCB-52、^13^C_12_-PCB-101、^13^C_12_-PCB-138、^13^C_12_-PCB-153、^13^C_12_-PCB-180)、PCN-MXA混合标准品(包括CN-13、CN-28、CN-52、CN-66、CN-73、CN-75)和PCN-MXC混合标准品(包括CN-27、CN-36、CN-46、CN-48、CN-50、CN-53、CN-69、CN-72)均购自加拿大Wellington Laboratories;4种^2^D标记的PCBs标准品(包括^2^D_4_-PCB-28、^2^D_6_-PCB-77、^2^D_4_-PCB-114、^2^D_3_-PCB-156)购自美国O2si公司。

### 1.2 采样和前处理材料的预处理

采样材料的预处理:购买未添加阻燃剂的聚氨酯泡沫(PUF),用85 ℃热水浸泡10 min后反复搓洗干净,沥干水分后放置通风橱内挥干残留的水分,之后用加速溶剂萃取仪进行清洗。清洗条件:溶剂为丙酮,温度为100 ℃,压力为10.34 MPa,静态时间为10 min,加热时间为5 min,循环2次,冲洗体积为70%萃取池体积,氮气吹扫60 s,重复萃取2次后,将PUF置于通风橱内挥干有机溶剂,并避光保存于干燥器中。石英纤维滤膜(QFF)于马弗炉中650 ℃高温焙烧4 h,处理好的滤膜用铝箔纸包好,置于干燥处密封保存,注意不可有折痕。

前处理材料的预处理:无水硫酸钠采用正己烷超声处理2次,每次10~20 min,随后转入陶瓷盘中,待溶剂挥发完全后保存在干燥器中。硅胶于马弗炉中650 ℃高温焙烧2 h以上,冷却后密封保存于干燥器中。

多层硅胶柱填料的制备:取98 g硅胶置于烧杯中,加入40 mL 50 g/L氢氧化钾溶液,充分搅拌,硅胶无结块后转移至圆底烧瓶,在50~80 ℃温度下减压脱水至粉末状,制备成质量分数2%的碱性硅胶。取100 g硅胶置于烧杯中,加入80 g浓硫酸,充分振荡至粉末状,制备成质量分数44%的酸化硅胶。取90 g硅胶置于烧杯中,加入28 mL 400 g/L硝酸银溶液,充分搅拌,硅胶无结块后转移至圆底烧瓶,在50~80 ℃温度下减压脱水至粉末状,制备成质量分数10%的硝酸银硅胶。制备好的填料均保存于干燥器中。

### 1.3 标准溶液配制

将1 μg/mL的PCNs标准品用壬烷配制成质量浓度为100 ng/mL的PCNs标准溶液。将10 μg/mL的^13^C_10_-CN-65用壬烷配制成质量浓度为1 μg/mL的PCNs采样内标溶液。将10 μg/mL的^13^C_10_-CN-13、^13^C_10_-CN-42、^13^C_10_-CN-27、^13^C_10_-CN-52、^13^C_10_-CN-67、^13^C_10_-CN-73、^13^C_10_-CN-75用壬烷配制成质量浓度为1 μg/mL的PCNs提取内标溶液。将10 μg/mL的^13^C_10_-CN-2和^13^C_10_-CN-64用壬烷配制成质量浓度为1 μg/mL的PCNs回收率内标溶液。

上述标准溶液均于-20 ℃冷藏保存。

### 1.4 样品的采集与保存

采样点设在中国科学院大连化学物理研究所生物楼楼顶平台(E 121°34.430', N 38°53.296',海拔60 m),大气采样仪距地面1.5 m,采样周围没有明显的局地污染。采样时间为2021年6~7月。使用QFF和PUF分别采集大气颗粒相和气相样品。采样前,先向PUF上添加10 ng采样内标,采样流速设置为800 L/min,连续采集6 h。实际采样体积以采样器显示值为准。采样结束后,将QFF和PUF分别用铝箔纸包裹,并于4 ℃密封保存。

### 1.5 样品前处理

样品提取和浓缩:分别在QFF和PUF上添加10 ng提取内标,进行加速溶剂萃取。萃取溶剂为二氯甲烷-正己烷(1:1, v/v),萃取温度为100 ℃,萃取压力为10.34 MPa,静态萃取时间为10 min,加热时间为5 min,萃取循环2次,冲洗体积为70%萃取池体积,氮气吹扫60 s。向萃取液中加入无水硫酸钠直至有流动的无水硫酸钠存在,静置30 min;然后将有机相转移至圆底烧瓶中,旋转蒸发至约2 mL。

浓缩液净化:浓缩液先过多层硅胶柱(自下而上依次装填无水硫酸钠2 g,硅胶1 g、2%碱性硅胶3 g、硅胶1 g、44%酸化硅胶8 g、硅胶1 g、10%硝酸银硅胶2 g和无水硫酸钠2 g),使用150 mL正己烷洗脱,洗脱液旋转蒸发至1~2 mL。该步骤所得浓缩液继续过中性氧化铝柱(自下而上依次装填无水硫酸钠2 g、中性氧化铝10 g和无水硫酸钠2 g),使用90 mL二氯甲烷-正己烷(5:95, v/v)进行洗脱,洗脱液旋转蒸发至1~2 mL,并将其转移至玻璃离心管中,用2 mL正己烷洗涤圆底烧瓶后合并至玻璃离心管中,在弱氮气流下吹干,加入10 ng回收率内标,壬烷定容至200 μL。

### 1.6 仪器分析

毛细管气相色谱柱为5%苯基-95%聚甲基硅氧烷(60 m×0.25 mm×0.25 μm),进样口温度为260 ℃,不分流进样,不分流时间为1 min。程序升温条件如下:初始温度80 ℃,保持1 min;以15 ℃/min的速率升温至160 ℃;再以3 ℃/min的速率升温至265 ℃;最后以5 ℃/min的速率升温至280 ℃,保持10 min。

质谱离子源为电子轰击(EI)源,传输线温度为280 ℃,离子源温度240 ℃。扫描方式为选择反应监测(SRM)模式,载气和碰撞气分别为高纯氦气和高纯氩气,定性、定量离子对和碰撞能参见表1。

**表 1:** PCNs和^13^C_10_-PCNs的保留时间、特征离子对及碰撞能

Item	Compound	Abbreviation		t_R_/min	Quantitative ion pair (m/z)	Qualitative ion pair (m/z)	IS	CE/eV
Target compound	1,4,6-tri-CN		CN-24	20.78	229.9/160.1	231.9/160.2	^13^C_10_-CN-13	27
	1,2,3-tri-CN		CN-13	22.22	229.9/160.1	231.9/160.2	^13^C_10_-CN-13	
	1,3,5,7-tetra-CN		CN-42	24.55	263.9/194.2	265.9/196.2	^13^C_10_-CN-42	29
	1,4,5,8-tetra-CN		CN-46	29.59	263.9/194.2	265.9/196.2	^13^C_10_-CN-27	
	1,2,3,5,7-penta-CN		CN-52	31.09	299.9/230.2	299.9/228.3	^13^C_10_-CN-52	29
	1,2,3,5,8-penta-CN		CN-53	33.86	299.9/230.2	299.9/228.3	^13^C_10_-CN-52	
	1,2,3,4,6,7-hexa-CN		CN-66	37.51	333.8/264.3	333.8/262.2	^13^C_10_-CN-67	29
	1,2,3,5,6,8-hexa-CN		CN-68	38.30	333.8/264.3	333.8/262.2	^13^C_10_-CN-67	
	1,2,3,4,5,6,7-hepta-CN		CN-73	44.37	367.8/298.3	367.8/296.3	^13^C_10_-CN-73	30
	1,2,3,4,5,6,7,8-octa-CN		CN-75	50.27	401.8/332.3	403.8/334.3	^13^C_10_-CN-75	31
Sampling standard	1,2,3,4,5,8-hexa-CN-^13^C_10_	^13^C_10_-	CN-65	40.58	343.8/274.3	345.8/274.3		29
Extraction standard	1,2,3-tri-CN-^13^C_10_	^13^C_10_-	CN-13	22.23	239.9/170.1	241.9/172.1	^13^C_10_-CN-2	27
	1,3,5,7-tetra-CN-^13^C_10_	^13^C_10_-	CN-42	24.54	273.9/204.2	275.9/196.2	^13^C_10_-CN-64	29
	1,2,3,4-tetra-CN-^13^C_10_	^13^C_10_-	CN-27	27.55	273.9/204.2	275.9/196.2	^13^C_10_-CN-64	
	1,2,3,5,7-penta-CN-^13^C_10_	^13^C_10_-	CN-52	31.09	309.9/240.2	307.9/238.2	^13^C_10_-CN-64	29
	1,2,3,5,6,7-hexa-CN-^13^C_10_	^13^C_10_-	CN-67	37.54	343.8/274.3	345.8/274.3	^13^C_10_-CN-64	29
	1,2,3,4,5,6,7-hepta-CN-^13^C_10_	^13^C_10_-	CN-73	44.39	377.8/308.3	379.8/308.3	^13^C_10_-CN-64	30
	1,2,3,4,5,6,7,8-octa-CN-^13^C_10_	^13^C_10_-	CN-75	50.30	411.8/342.3	413.8/344.3	^13^C_10_-CN-64	31
Recovery standard	2-mono-CN-^13^C_10_	^13^C_10_-	CN-2	12.38	171.9/137.1	173.9/137.1		25
	1,2,3,4,5,7-hexa-CN-^13^C_10_	^13^C_10_-	CN-64	38.29	343.8/274.3	345.8/274.3		29

CN: chloronaphthalene.

## 2 结果与讨论

### 2.1 方法优化

#### 2.1.1 气相色谱条件优化

目前仅有国际标准化组织发布了关于水质中多氯萘测定的气相色谱-质谱法标准操作流程(ISO TS 16780-2015)。在该标准建议的色谱条件下,本研究通过分析多氯萘标准品和工业产品对程序升温进行了优化,使得所有多氯萘异构体都得到了较好的分离。进样口温度的设定是色谱分析的关键条件之一,进样口温度过低会导致目标化合物不能完全气化,过高则会导致目标物分解。本研究在选择反应监测模式下,对250、260和270 ℃进样口温度进行优化。从图2可以看出,多氯萘在进样口温度为260 ℃条件下,信号响应强度最高。因此,最佳进样口温度确定为260 ℃。

**图 2:**
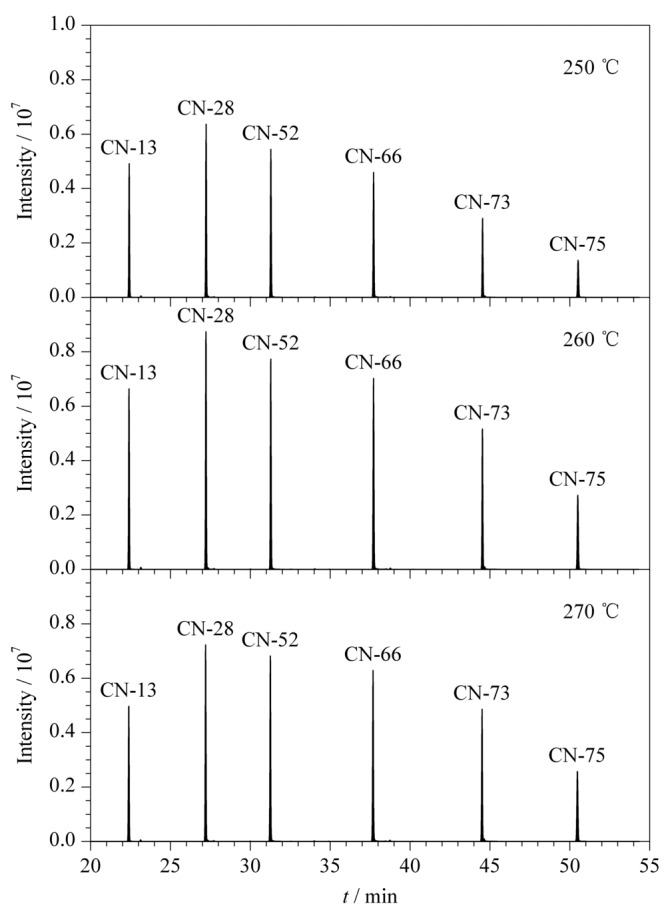
进样口温度对PCNs(5 μg/mL)信号响应强度的影响

#### 2.1.2 质谱条件优化

首先将PCNs标准品溶液进行全扫描(Full Scan),获得质荷比较大且相对丰度较高的特征碎片离子作为母离子。然后采用SRM模式,对可能产生的子离子进行全扫描,确定丰度比较大的两个离子作为子离子,与母离子形成特征离子对。设定碰撞能25、26、27、28、29、30、31、32、33、34、35、36、37、38、39和40 eV序列,进行碰撞能优化,使子离子碎片质谱响应值最大。PCNs标准物质的特征离子对和优化后的最佳碰撞能见表1。

离子源温度的高低会影响离子化效率,从而对多氯萘信号响应强度产生直接的影响。本实验分别考察了离子源温度为200、220、240和260 ℃时,PCNs信号响应强度的变化。从图3可以看出,不同氯代同类物的信号响应强度随离子源温度升高呈现先增大后减小的趋势,离子源温度选择240 ℃时,可以得到最大的响应值。因此最佳离子源温度确定为240 ℃。

**图 3:**
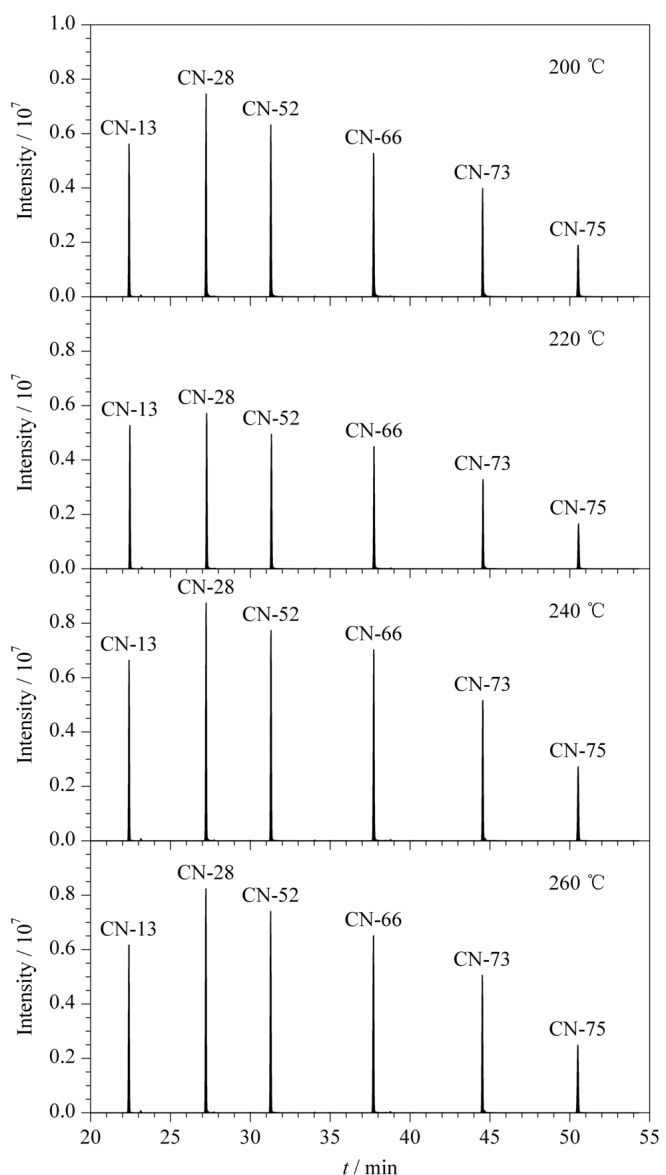
离子源温度对PCNs(5 μg/mL)信号响应强度的影响

最佳的GC-MS/MS条件下,PCNs同类物的SRM总离子流色谱图如图4。

**图 4:**
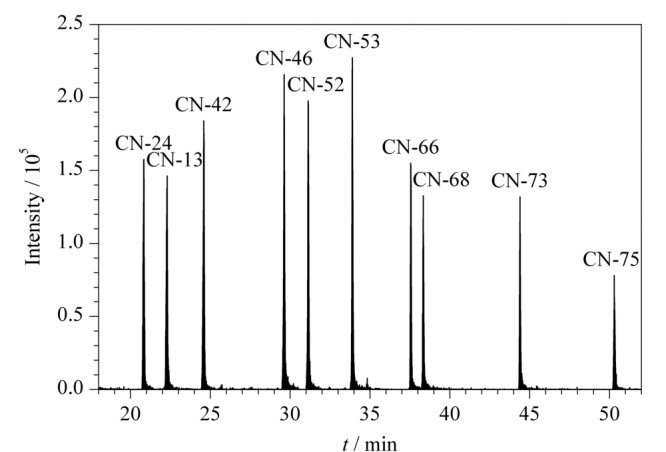
PCNs标准溶液(100 ng/mL)在选择反应监测 模式下的总离子流色谱图

#### 2.1.3 提取方法优化

多氯萘常用的提取溶剂主要包括甲苯、丙酮、正己烷、二氯甲烷或其混合溶剂等^[7,8,12,18]^。考虑到甲苯沸点较高,浓缩较为困难,因此主要考察正己烷和二氯甲烷或丙酮的混合溶剂,主要包括二氯甲烷-正己烷(1:4, v/v)、二氯甲烷-正己烷(1:1, v/v)、丙酮-正己烷(1:4, v/v)和丙酮-正己烷(1:1, v/v)。为了避免样品本底值对实验结果的影响,本实验以^13^C_10_-CN-13、^13^C_10_-CN-42、^13^C_10_-CN-27、^13^C_10_-CN-52、^13^C_10_-CN-67、^13^C_10_-CN-73、^13^C_10_-CN-75作为研究对象,^13^C_10_-CN-2和^13^C_10_-CN-64作为内标。研究发现:经丙酮-正己烷(1:1, v/v)提取浓缩后,管壁有白色固体附着。从图5可以看出,不同体积比的混合溶剂的提取效率差别不大。结合实验现象和相关文献报道^[7,9,12]^,二氯甲烷和正己烷的混合溶剂对PUF中多氯萘提取效果较好,最终,选用二氯甲烷-正己烷(1:1, v/v)混合溶剂作为提取溶剂。

**图 5:**
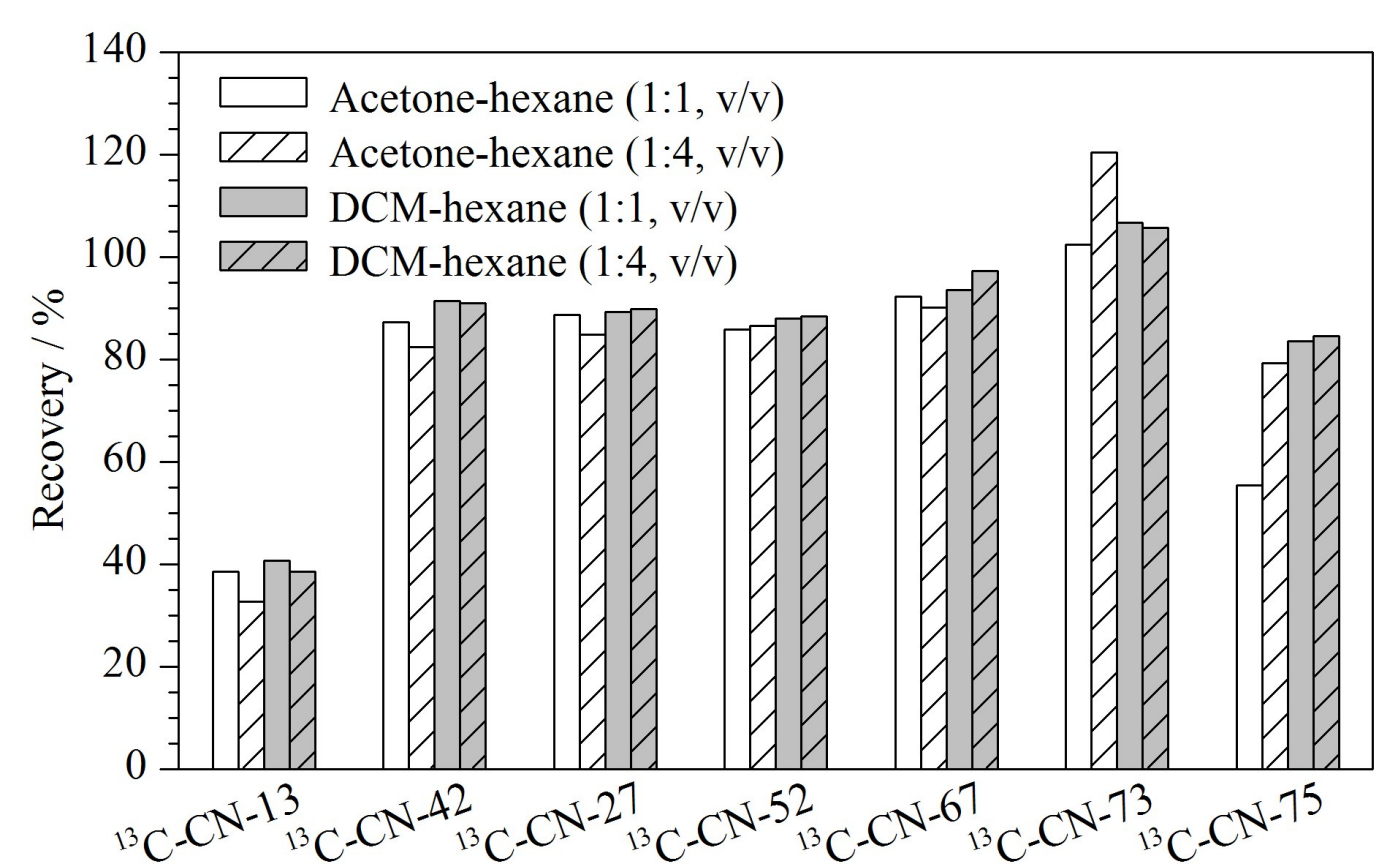
提取溶剂对^13^C_10_-PCNs提取效率的影响

#### 2.1.4 净化方法优化

为了确保多氯萘在净化过程中的有效回收,本研究采用多层硅胶和中性氧化铝柱联合净化,分别考察了多氯萘在两种柱色谱上的流出曲线。10 ng PCNs标准物质分别用2 mL正己烷稀释后作为上样溶液,其中一份过多层硅胶柱,以250 mL正己烷溶液进行洗脱,每50 mL为一个馏分,共收集5个馏分,记作F_1_~F_5_;另一份过中性氧化铝柱,以150 mL二氯甲烷-正己烷(5:95, v/v)溶液进行洗脱,每30 mL为一个馏分,共收集5个馏分,记作F_6_~F_10_。研究发现:在多层硅胶柱上,多氯萘主要集中在F_2_和F_3_两个馏分被洗脱下来;在中性氧化铝柱上,多氯萘主要集中在F_7_和F_8_两个馏分被洗脱下来。为了确保目标物洗脱完全,最终采用150 mL正己烷和90 mL二氯甲烷-正己烷(5:95, v/v)分别作为两个柱色谱上多氯萘的洗脱溶剂。

### 2.2 采样过程中的吸附和穿透实验

由于采集后的实际样品本底值对实验结果会产生影响,因此采用^13^C_10_-PCNs标准物质作为分析对象,但现有的^13^C_10_-PCNs标准物质数量有限,为了能够全面考察不同氯取代萘在采样过程中的回收率情况,本研究采用与其结构性质都相近的^13^C_12_-PCBs进行代替。采用上下串联两个预处理后的PUF(PUF 1和PUF 2)进行气相样品的采集,样品采集前向PUF 1中加入10 ng采样内标(^13^C_10_-CN-13、^13^C_12_-PCB-28、^13^C_12_-PCB-52、^13^C_12_-PCB-101、^13^C_12_-PCB-138、^13^C_12_-PCB-153、^13^C_10_-CN-65和^13^C_12_-PCB-180),分别以800 L/min的流量主动采集6 h和24 h后,按照样品分析的全部步骤进行测定。同位素标记的PCBs的特征离子对和碰撞能参考表2。

**表 2:** ^13^C_12_-PCBs和^2^D-PCBs的保留时间、特征离子对及碰撞能

Compound	Abbreviation		t_R_/min	Quantitative ion pair (m/z)	Qualitative ion pair (m/z)	IS		CE/eV
2,4,4'-Tri-PCB-^13^C_12_	^13^C_12_-	PCB-28	24.66	268.0/198.0	270.0/198.0	^2^D_4_-	PCB-28	35
2,2',5,5'-Tetra-PCB-^13^C_12_	^13^C_12_-	PCB-52	26.59	304.0/234.0	304.0/232.0	^2^D_6_-	PCB-77	34
2,2',4,5,5'-Penta-PCB-^13^C_12_	^13^C_12_-	PCB-101	31.70	338.0/268.0	338.0/303.0	^2^D_4_-	PCB-114	35
2,2',3,4,4',5-Hexa-PCB-^13^C_12_	^13^C_12_-	PCB-138	36.70	372.0/302.0	372.0/300.0	^2^D_3_-	PCB-156	36
2,2',4,4',5,5'-Hexa-PCB-^13^C_12_	^13^C_12_-	PCB-153	38.35	372.0/302.0	372.0/300.0	^2^D_3_-	PCB-156	36
2,3,3',4,4',5,5'-Hepta-PCB-^13^C_12_	^13^C_12_-	PCB-180	42.22	406.0/336.0	406.0/334.0	^2^D_3_-	PCB-156	37
2,4,4'-Tri-PCB-^2^D_4_	^2^D_4_-	PCB-28	24.60	260.0/190.0	262.0/190.0			36
3,3',4,4'-Tetra-PCB-^2^D_6_	^2^D_6_-	PCB-77	33.89	298.0/228.0	298.0/226.0			38
2,3,4,4',5-Penta-PCB-^13^C_12_	^2^D_4_-	PCB-114	36.07	330.0/260.0	330.0/258.0			44
2,3,3',4,4',5-Hexa-PCB-^2^D_3_	^2^D_3_-	PCB-156	41.31	363.0/293.0	363.0/291.0			44

从表3中可以看出,采样时间为6 h时,PUF 1中同位素标记的三氯萘和三氯联苯(^13^C_10_-CN-13和^13^C_12_-PCB-28)的采样内标回收率为52.5%~72.2%,目标物未穿透第一个PUF,随采样时间的增加,采样内标回收率降低;采样时间为6 h时,PUF 1中同位素标记的4~7氯联苯和六氯萘采样内标回收率可达75.3%~112.6%,当采样时间增加至24 h也未发生穿透现象。因此,超大流量采样6 h,可实现对三氯以上多氯萘的定量采集,对于四氯以上多氯萘而言,采样时间增加至24 h,也不会对采样回收率产生明显影响。

**表 3:** 不同采样时间时^13^C_10_-PCNs和^13^C_12_-PCBs的采样回收率

Compound	PUF 1 (6 h)	PUF 2 (6 h)	PUF 1 (24 h)	PUF 2 (24 h)
^13^C_10_-CN-13	52.5	0.5	35.4	0.3
^13^C_12_-PCB-28	72.2	0.1	62.1	0.1
^13^C_12_-PCB-52	75.3	-	69.1	0.2
^13^C_12_-PCB-101	92.3	-	92.5	0.2
^13^C_12_-PCB-138	89.5	-	90.5	0.1
^13^C_12_-PCB-153	74.4	-	79.1	0.1
^13^C_10_-CN-65	112.6	0.2	104.1	1.7
^13^C_12_-PCB-180	84.0	-	79.6	0.2

-: no data.

### 2.3 方法适用性验证

#### 2.3.1 平均相对响应因子和仪器检出限

将PCNs混合标准溶液分别配制成质量浓度为2、5、10、20、50、100 ng/mL的系列标准溶液,内标溶液的质量浓度为50 ng/mL,运用已建立的GC-MS/MS方法对上述标准溶液进行3次重复测定,按照如下公式(1),分别计算3~8氯萘的平均相对响应因子(RRF)及其相对标准偏差(RSD)。结果表明,在2~100 ng/mL范围内,PCNs的平均相对响应因子的相对标准偏差均小于16%(见表4)。选择系列标准溶液中最低浓度的标准溶液进行5次以上重复测定,计算测定值的标准偏差,取标准偏差的3倍作为仪器检出限(IDLs),结果见表4。

**表 4:** PCNs的平均相对响应因子、相对标准偏差、仪器检出限和方法检出限(MDL)

Compound	Average RRF	RSD/%	IDL/(pg)	MDL/(pg/m^3^)
CN-24	0.75	11.9	1.0	1
CN-13	0.76	7.3	0.7	1
CN-42	1.11	10.5	0.8	3
CN-46	0.95	9.8	0.9	2
CN-52	0.89	14.6	0.9	3
CN-53	0.85	15.9	0.9	2
CN-66	0.85	9.8	1.0	3
CN-68	0.73	11.2	0.9	2
CN-73	0.94	11.4	0.7	2
CN-75	0.84	15.2	0.9	3


(1) $RRF=\frac{Q_{es}}{Q_s}\times \frac{A_s}{A_{es}}$

式中:*Q*_es_代表标准溶液中提取内标物质的绝对量(pg); *Q*_s_代表标准溶液中待测化合物的绝对量(pg); *A*_s_代表标准溶液中待测化合物的监测离子峰面积;*A*_es_代表标准溶液中提取内标物质的监测离子峰面积。

#### 2.3.2 方法检出限

方法检出限参考《环境监测分析方法标准制定技术指导》(HJ 168-2020)^[19]^中规定的方法进行评估。使用与实际采样操作相同的采样材料作为试样,按照样品分析的全部步骤,重复7次空白试验;对于空白试验中检测出的目标物(CN-24和CN-13),将各测定结果换算为样品中的含量(以样品体积288 m^3^计),计算7次平行测定的标准偏差,取标准偏差的3.143倍作为方法检出限;对于空白试验中未检出的目标物(CN-42、CN-46、CN-52、CN-53、CN-66、CN-68、CN-73和CN-75),向PUF中添加1.0 ng PCNs标准物质,按照样品分析的全部步骤,重复7次平行实验,将各测定结果换算为样品中的含量(以样品体积288 m^3^计),计算7次平行测定的标准偏差,取标准偏差的3.143倍作为方法检出限,最终确定大气样品的方法检出限为1~3 pg/m^3^,具体结果见表4。

#### 2.3.3 精密度和准确度

为保障方法验证样品的统一性。样品采集时向预处理后的PUF中加入7×10 ng采样内标,采集6 h后,将PUF和QFF进行加速溶剂提取,合并提取液并按照称重法平均分成7份,其中一份仅添加提取内标量,另外6份分别加入等量的目标物标准溶液和提取内标(10 ng),然后按照本方法的浓缩、净化和分析全程序平行测定7次,添加的目标物标准溶液分为低、中、高(20、50、90 ng/mL)3个水平。

从表5可以看出,低、中、高加标水平下PCNs的平均回收率范围分别为89.0%~119.4%、98.6%~122.5%和93.7%~124.5%, RSD分别为1.9%~7.0%、1.6%~6.6%和1.0%~4.8%。整个分析过程中,采样内标和提取内标的平均回收率分为136.2%~146.0%和42.4%~78.1%, RSD分别为5.6%~7.5%和2.7%~17.5%,满足痕量分析的要求^[20]^,且平行性较好。该方法的准确度高,精密度好。

**表 5:** 环境空气中多氯萘的平均加标回收率和相对标准偏差(*n*=6)

Compound	Background/ (ng/mL)	Added/ (ng/mL)	Found/ (ng/mL)	Recovery/ %	RSD/ %	Compound	Background/ (ng/mL)	Added/ (ng/mL)	Found/ (ng/mL)	Recovery/ %	RSD/ %
CN-24	7.4	20.0	31.2	119.4	6.6	CN-42	0.3	20.0	20.5	101.3	7.0
	9.9	50.0	64.7	109.5	4.5		0.2	50.0	51.8	103.1	3.4
	8.0	90.0	107.2	110.2	4.8		0	90.0	92.6	102.8	4.0
CN-13	5.2	20.0	28.3	115.4	5.9	CN-46	0.8	20.0	24.1	116.7	4.3
	6.6	50.0	64.2	115.3	3.9		0.5	50.0	61.7	122.5	4.1
	5.3	90.0	116.6	123.7	2.6		0	90.0	112.1	124.5	4.3
CN-52	0.4	20.0	21.0	103.2	3.0	CN-68	0.5	20.0	21.7	106.1	5.3
	0.2	50.0	54.6	108.8	1.6		0.5	50.0	58.3	115.7	6.6
	0	90.0	92.7	103.0	4.8		0	90.0	102.2	113.5	1.0
CN-53	0.5	20.0	20.9	102.2	1.9	CN-73	1.5	20.0	19.3	89.0	3.7
	0.4	50.0	53.0	105.2	1.9		0.6	50.0	49.9	98.6	2.6
	0.1	90.0	99.2	110.1	2.6		0	90.0	84.3	93.7	3.4
CN-66	1.0	20.0	22.6	107.9	5.0	CN-75	1.3	20.0	19.5	91.3	5.5
	0.1	50.0	55.5	110.6	3.6		0.2	50.0	51.2	101.9	3.8
	0	90.0	102.9	114.4	1.9		0	90.0	98.5	109.4	3.5

## 3 结论

本文优化了样品前处理过程和仪器分析中的重要参数,建立了加速溶剂萃取结合多层硅胶/中性氧化铝层析柱提取、净化环境空气中PCNs的前处理方法,并采用同位素稀释气相色谱-三重四极杆质谱法对PCNs进行定性和定量分析。适用性验证结果表明:该方法具有较低的方法检出限和较高的准确度和精密度,可用于环境空气样品中3~8氯萘的准确测定。不仅如此,本方法可在一定程度上缓解多氯萘监测对高分辨气相色谱-高分辨质谱的依赖,为实现国际履约提供方法支持。
